# Investigating the Association Between Cystic Fibrosis and Colorectal Neoplasia: A Matched Case–Control Study

**DOI:** 10.1002/jgh3.70440

**Published:** 2026-07-14

**Authors:** Dazhong Huang, Douglas Tjandra, Ammar Majeed, Gregory Snell, Stuart K. Roberts, Gregor Brown, Alex Boussioutas, Peter Wark, Shara Ket

**Affiliations:** ^1^ Department of Gastroenterology and Hepatology The Alfred Hospital Melbourne Australia; ^2^ Department of Respiratory Medicine and Lung Transplantation The Alfred Hospital Melbourne Australia; ^3^ Department of Gastroenterology School of Translational Medicine, Monash University Melbourne Australia

**Keywords:** cancer screening, colorectal neoplasia, cystic fibrosis

## Abstract

**Background:**

Cystic fibrosis (CF) is an autosomal recessive disorder that has been associated with increased risk of colorectal neoplasia and cancer (CRC). Current consensus statements recommend early screening and surveillance colonoscopies for CRC in CF, though high‐quality data supporting this remains lacking.

**Methods:**

We performed a retrospective case–control study in a statewide CF referral center, comparing patients with CF and matched controls who underwent elective colonoscopy between 2010 and 2024.

**Results:**

Two hundred and forty‐five patients with CF were included and matched 1:1 to control cohort. Significantly higher number of patients in CF cohort had neoplastic lesions (51.8% vs. 24.1%, OR 3.41, 95%‐CI 2.32–5.02, *p* < 0.0001), advanced lesions (16.3% vs. 10.2%, OR 1.76, 95%‐CI 1.03–2.99, *p* = 0.039), and CRC (4.9% vs. 0.8%, OR 6.26, 95%‐CI 1.39–28.26, *p* = 0.017). CRC‐free survival was significantly lower in CF (log‐rank *p* = 0.007). Age and lung transplant recipients were predictors on multivariate analysis for colorectal neoplasia. Incidence of CRC in asymptomatic patients with CF at initial colonoscopy was 5.5% and mean age of diagnosis was 40.5 years.

**Conclusions:**

This study highlights the increased association of CF with colorectal neoplasia and CRC, and the need for careful screening and surveillance via colonoscopy.

## 1 Introduction

Cystic fibrosis (CF) is a disorder arising from mutations in the CF transmembrane conductance regulator (CFTR) gene that normally regulates anion channel function of epithelial cells in multiple organs [1, 2, 3, 4]. This results in the production of thickened mucus that obstructs airways, leading to impaired lung function and recurrent infection. It may also cause impaired immunity, reduced intestinal motility and constipation, pancreatic insufficiency, impaired renal function, etc. [1, 3, 4, 5, 6, 7, 8].

CF is also associated with increased cancer risk, especially colorectal cancer (CRC) [9, 10, 11, 12, 13]. In a matched case–control study at our unit, Gory et al. found a 10‐fold higher incidence of CRC [9]. Consensus recommendations by Hadjiliadis et al. recommend commencing CRC screening via colonoscopy from age 40 for CF patients, and 30 in those with solid organ transplant within 2 years of recovery from transplantation [14]. Surveillance intervals of ≤ 5 years are recommended if screening colonoscopy found no neoplastic lesions and ≤ 3 years intervals with lesion detection [14]. However, there is a lack of high‐quality studies analyzing the incidence of colorectal neoplasia and CRC in CF to support these statements.

## 2 Materials and Methods

This is a retrospective single‐center observational case–control study to determine the incidence of colorectal neoplastic lesions found in patients with CF during elective colonoscopy. Patients with CF were identified from the Alfred Cystic Fibrosis database in collaboration with the Alfred Hospital Cystic Fibrosis Service in Melbourne, the largest statewide referral center for CF in Australia. Inclusion criteria are adults aged ≥ 18 years with diagnosed CF, who underwent elective colonoscopy at the Alfred Hospital between January 2010 and December 2024. Those aged < 18 years, with hereditary colonic polyposis syndromes, active inflammatory bowel disease, or who underwent flexible sigmoidoscopy or emergency inpatient colonoscopy were excluded.

Using propensity score matching based on age and gender in 1:1 ratio, a control cohort of individuals without CF who underwent elective colonoscopy at the Alfred Hospital during the study period was obtained. Controls were not matched for lung function or immunosuppressed status. Control patients with hereditary colonic polyposis syndrome, active inflammatory bowel disease, or those who underwent flexible sigmoidoscopy or emergency inpatient colonoscopy were excluded.

Data on baseline demographics, disease characteristics, treatment history, and histopathology reports were obtained through electronic medical records available on Cerner Powerchart (Oracle Health, Kansas City, Missouri, USA). Data on colonoscopy indication, bowel preparation quality, colorectal neoplastic lesion size, number, and location were obtained through ENDOBASE Endoscopy Documentation Solution (Olympus, Shinjuku, Japan).

Bowel preparation protocols for CF patients at the Alfred Hospital had been repeatedly modified during the study period. As of 2024, it consists of an intensive regimen of four daily Macrogol 3350 (Movicol) sachets and four daily Bisacodyl tablets over the preceding 7 days, a white diet over 3 days and split‐regimen Moviprep (PEG‐3350, sodium sulfate, sodium chloride, potassium chloride, sodium ascorbate, and ascorbic acid in 2 L solution) and Plenvu (PEG‐3350, sodium ascorbate, sodium sulfate, ascorbic acid, sodium chloride, and potassium chloride in 1 L solution) over 2 days prior to colonoscopy. Bowel preparation for the control cohort consisted of standard regimens of 2 days of white diet and 2 L of split‐regimen Moviprep. In patients with previous or anticipated poor preparation quality, they additionally received 7 days of two daily sachets of Macrogol 3350 (Movicol).

Primary study outcome is the incidence of colorectal neoplastic lesions detected during colonoscopy in both CF and matched control cohorts. Neoplastic lesions were defined as adenomas, sessile serrated lesions, traditional serrated lesions, or adenocarcinoma based on histopathological assessment of resected lesions. Nonneoplastic lesions including hyperplastic or inflammatory polyps were excluded. Secondary outcomes include adequacy of bowel preparation, incidence of advanced colorectal neoplastic lesions (defined as lesion size ≥ 10 mm, or presence of villous features, high grade dysplasia, or adenocarcinoma), CRC, size of largest lesion detected in each colonoscopy, and lesion number per colonoscopy.

### 2.1 Statistical Analysis

Normality was assessed using descriptive statistics, normal probability plots, and/or Shapiro–Wilks test. Descriptive statistics were presented as mean ± standard deviation (SD) for normally distributed variables, median (interquartile range [IQR]) for nonnormally distributed variables, and proportions (percentages) as appropriate. Independent samples *t*‐tests were used to analyze normally distributed numerical variables. Wilcoxon rank‐sum tests were used for nonnormal variables. Chi‐squared tests were used to analyze categorical variables when observed frequencies in each cell were ≥ 5. CRC‐free survival was analyzed with Kaplan–Meier curves. Univariable and multivariable analyses were performed using logistic regression; factors with *p* ≤ 0.05 were included in multivariate analysis. For logistic regression analysis, due to wide variety of colonoscopy indications documented, indications for colonoscopy were grouped using a hierarchy of severity into screening for CRC with no symptoms, low‐risk symptoms only (altered bowel habit and abdominal pain/bloating), one higher risk symptoms/indications only (iron deficiency anemia, GI bleeding, family history CRC, FOBT positivity, weight loss, and polyp surveillance), and two or more high‐risk symptoms/indications [15]. When multiple indications were recorded for a single colonoscopy, the indication of the “highest” severity category was recorded. Multivariate analysis was not performed among those with CRC due to low numbers in both cohorts. All *p*‐values were two‐tailed and results were considered statistically significant if *p* < 0.05. Analyses were conducted in STATA and/or SPSS statistical software.

## 3 Results

**FIGURE 1:**
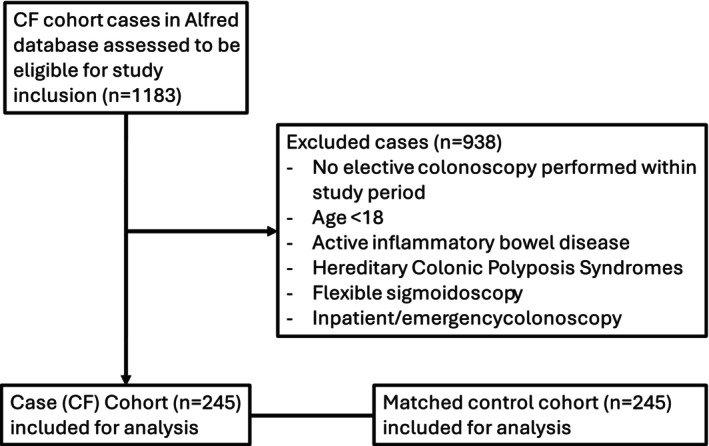
CONSORT diagram of CF and control cohort study participation.

From January 2010 to December 2024, 245 CF patients underwent 556 elective colonoscopies and were matched with 245 control patients who underwent 318 elective colonoscopies (Figure 1). Number of colonoscopies in CF patients per year is shown in Figure S1. Baseline demographics and initial colonoscopy indications are summarized in Table 1. In both cohorts, mean age was 43 years and 58% were male. Among CF cohort, 107/245 (43.7%) were previous lung transplant recipients, with a median of 13 years since transplant.

**TABLE 1:** Baseline demographics and indications for colonoscopy.

	CF cases (*n* = 245)	Control cases (*n* = 245)	*p*
Baseline demographics			
Mean age (±SD)	43 ± 10.1	43 ± 10.1	
Male sex	142 (58%)	142 (58%)	
Lung transplant recipient	107 (43.7%)	0	
Median ASA score	3	2	
Indications (initial colonoscopy)			
Family history of CRC	23 (9.4%)	25 (10.2%)	0.76
Iron deficiency	63 (25.7%)	72 (29.4%)	0.36
Altered bowel habit	45 (18.4%)	86 (35.1%)	< 0.01
Abdominal pain/bloating	9 (3.7%)	12 (4.9%)	0.50
Rectal bleeding	28 (11.4%)	31 (12.7%)	0.68
Weight loss	24 (9.8%)	20 (8.2%)	0.53
Fecal occult blood test (FOBT)	13 (5.3%)	20 (8.2%)	0.21
CF screen/surveillance	86 (35.1%)	—	—
Surveillance of previous polyps	8 (3.6%)	11 (4.5%)	0.48

Abbreviations: ASA: American Society of Anesthesiologists; CF: cystic fibrosis; CRC: colorectal cancer.

Mean age at initial colonoscopy was similar in both cohorts (36.8 vs. 39.1 years respectively). Median American Society of Anesthesiologists (ASA) score was 3 in CF cohort and 2 in control cohort.

### 3.1 Bowel Preparation

**FIGURE 2:**
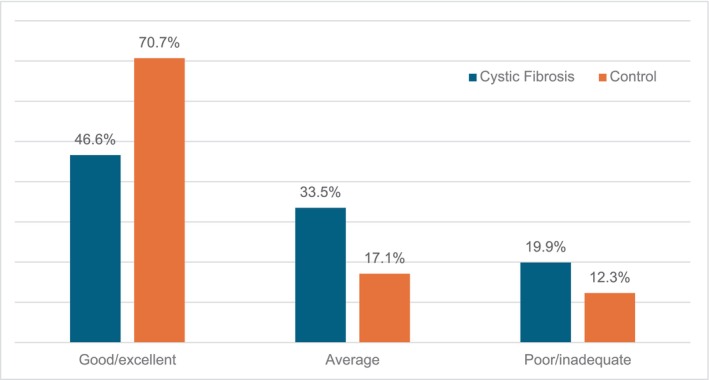
Quality of bowel preparation in CF and control cohorts.

Bowel preparation quality was documented in 547/556 colonoscopies in CF cohort and 293/318 in controls (Figure 2). Among all colonoscopies, the CF cohort had a lower proportion with good/excellent bowel preparation (46.6% vs. 70.7%, OR 0.36, 95%‐CI 0.27–0.49, *p* < 0.01), and a higher proportion with poor bowel preparation (19.9% vs. 12.3%, OR 1.78, 95%‐CI 1.18–2.67, *p* < 0.01).

### 3.2 Colorectal Neoplastic Lesions

Rate of detection of colorectal neoplastic lesions in both cohorts is summarized in Tables 2 and 3.

**TABLE 2:** CF versus control cohort.

Outcomes	CF (*n* = 245)	Control (*n* = 245)	OR (95% CI)	*p*
Patients with colonic neoplasia detected				
Initial colonoscopy	77 (31.42%)	50 (20.41%)	1.79 (1.18–2.69)	< 0.01
Among total colonoscopies	127 (51.84%)	59 (24.08%)	3.41 (2.32–5.01)	< 0.01
Patients with advanced neoplastic lesions				
Initial colonoscopy	32 (13.06%)	18 (7.35%)	2.01 (1.09–3.74)	0.03
Among total colonoscopies	40 (16.32%)	25 (10.20%)	1.76 (1.03–2.99)	0.04
Patients with adenocarcinoma				
Initial colonoscopy	8 (3.27%)	1 (0.41%)	8.24 (1.02–66.36)	< 0.05
Among total colonoscopies	12 (4.9%)	2 (0.8%)	6.26 (1.39–28.26)	0.02

Abbreviations: CF: cystic fibrosis; OR: unadjusted odds ratio.

**TABLE 3:** Outcomes per colonoscopy in CF versus control cohort.

Outcomes per colonoscopy	CF (*n* = 556)	Control (*n* = 318)	OR (95% CI)	*p*
Good/excellent bowel preparation	255 (46.6%)	207 (70.7%)	0.36 (0.27–0.49)	< 0.01
Colonoscopies with colonic neoplasia detected	202 (36.3%)	69 (21.7%)	2.06 (1.50–2.83)	< 0.01
Colonoscopies with advanced neoplastic lesions	48 (8.6%)	27 (8.5%)	1.02 (0.62–1.67)	0.94
Colonoscopies with adenocarcinoma	14 (2.5%)	2 (0.6%)	4.08 (0.92–18.07)	0.06

Abbreviations: CF: cystic fibrosis; OR: unadjusted odds ratio.

A higher proportion of patients in the CF cohort had neoplastic lesions found on initial colonoscopy than the control cohort (77 vs. 50 respectively, OR 1.79, 95%‐CI 1.18–2.69, *p* < 0.01). Similarly, a higher proportion in the CF cohort had advanced lesions detected on initial colonoscopy than the control (32 vs. 18 respectively, OR 2.01, 95%‐CI 1.09–3.74, *p* = 0.03).

A higher proportion of patients in CF cohort had colorectal neoplastic lesions detected across all colonoscopies compared to control cohort (51.8% vs. 24.1%, OR 3.41, 95%‐CI 2.32–5.01, *p* < 0.01). Similarly, higher proportion of all colonoscopies performed in CF cohort detected neoplastic lesions (36.3% vs. 21.7%, OR 2.06, 95%‐CI 1.50–2.83, *p* < 0.01). Higher proportion of patients in CF cohort had advanced neoplastic lesions (16.3% vs. 10.2%, OR 1.76, 95%‐CI 1.03–2.99, *p* = 0.04), but no difference was seen between cohorts in proportion of all colonoscopies that detected advanced lesions. No difference was seen in mean largest lesion size (8.5 ± 10.4 vs. 7.7 ± 6.9 mm, *p* = 0.26) or lesion number (median two lesions in both cohorts).

**FIGURE 3:**
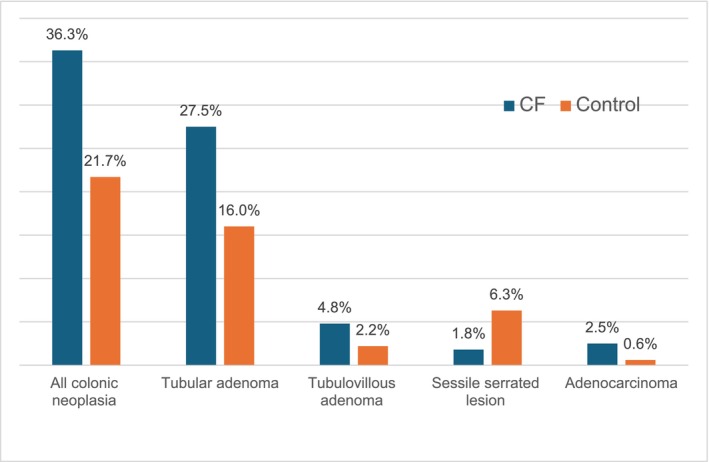
Proportion of all colonoscopies with neoplastic lesions detected.

Figure 3 summarizes the proportion of neoplastic lesion histopathology detected in all colonoscopies in both cohorts. In CF cohort, tubular adenomas represented a higher proportion of all neoplastic lesions (75.7% vs. 62.5% of all colonoscopies performed, OR 1.78, 95%‐CI 1.02–3.10, *p* = 0.04). Conversely, sessile serrated lesions represented a lower proportion of all neoplastic lesions (4.95% vs. 25%, OR 0.16, 95%‐CI 0.07–0.35, *p* < 0.01). Proportion of tubulovillous adenomas did not differ (13.40% vs. 8.75%, OR 1.61, 95%‐CI 0.67–3.86, *p* = 0.29).

Mean age of patients in CF cohort with detected neoplastic lesions at initial colonoscopy was younger compared to control cohort (41.4 vs. 45.4 years, *p* < 0.03). Age and previous lung transplant were associated with neoplastic lesion detection on univariate and multivariate analysis (Table 4), while only delf508 homozygosity was predictive of advanced lesions on univariate analysis (OR 17.42, 95%‐CI 2.25–134.6, *p* < 0.01, Table S1), though genotype data was not available for 37% of the CF cohort. Comparison of detection of neoplastic lesions, advanced lesions, and cancer in transplanted, nontransplant CF patients, and controls is shown in Figure S2.

**TABLE 4:** Logistic regression analysis of predictors for neoplastic lesions in CF cohort.

Variables	Univariate analysis		Multivariate analysis	
	OR (95% CI)	*p*	OR (95% CI)	*p*
Age (per decade)	1.70 (1.29–2.25)	< 0.01	1.67 (1.27–2.20)	< 0.01
Gender (reference: male)	1.30 (0.78–2.17)	0.31		
CF mutation (reference: heterozygous)	1.48 (0.74–2.95)	0.26		
Lung transplant (reference: no transplant)	1.80 (1.08–3.00)	0.02	1.73 (1.02–2.92)	0.04
Time since transplantation (years)	1.03 (0.97–1.09)	0.35		
Family history of CRC	1.53 (0.64–3.63)	0.34		
Colonoscopy indication (reference: screen for CRC only without symptoms)				
Low‐risk symptoms only	1.18 (0.53–2.65)	0.68		
One higher risk symptom/indication	0.81 (0.45–1.44)	0.46		
Two or more higher risk symptoms/indications	2.65 (0.48–14.57)	0.26		
Presence of intestinal symptoms	0.91 (0.54–1.54)	0.73		
ASA (reference: ASA 2)				
ASA 3	1.39 (0.73–2.67)	0.31		
ASA 4	2.18 (0.71–6.72)	0.18		
Bowel preparation quality (reference: good/excellent)				
Average	0.89 (0.49–1.58)	0.68		
Poor	1.51 (0.77–2.96)	0.28		

Abbreviations: ASA: American Society of Anesthesiologists physical assessment score; CF: cystic fibrosis; CRC: colorectal cancer; OR: unadjusted odds ratio.

### 3.3 Cancer

**FIGURE 4:**
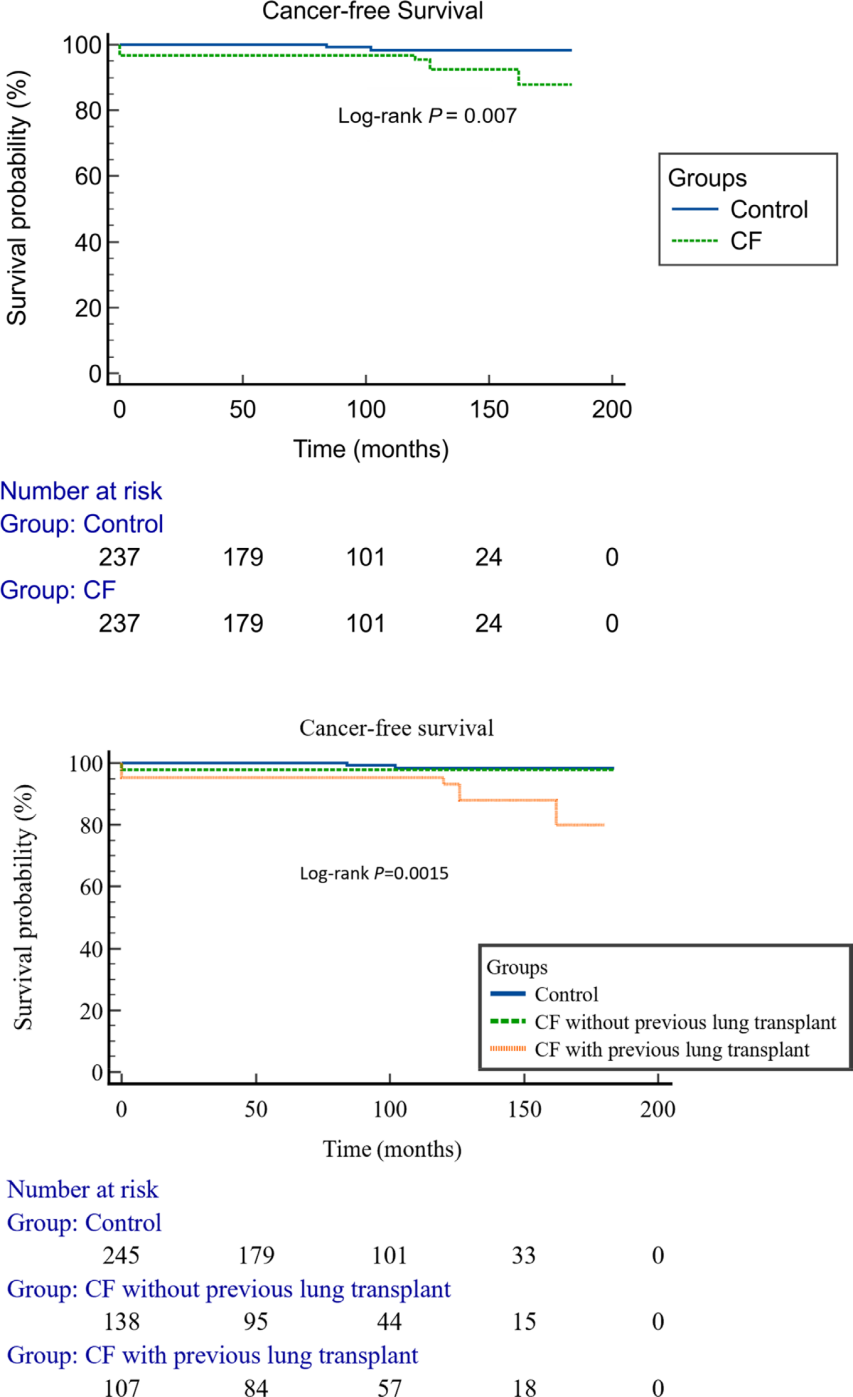
Cancer‐free survival in CF versus control cohorts (top) and CF with lung transplant, without lung transplant versus control cohort (bottom).

Twelve patients in CF cohort and two in control cohort developed CRC. In CF cohort, eight patients were diagnosed with cancer on initial colonoscopy (mean age 42.8 years) and four were diagnosed during surveillance (median 3 years after initial colonoscopy). Among asymptomatic patients in CF cohort undergoing screening colonoscopy for CRC, four patients (5.5%) were diagnosed with CRC at initial colonoscopy at mean age of 40.5 years. In the control cohort, one patient was diagnosed at initial screening colonoscopy and one diagnosed during surveillance 4 years after initial colonoscopy. Two CF patients developed cancer recurrence detected on surveillance colonoscopy after previous surgical resection. Nine out of 12 CF patients with CRC were lung transplant recipients. CRC occurred in higher proportion of CF cohort (4.9% vs. 0.8%, OR 6.26, 95%‐CI 1.39–28.26, *p* < 0.02), especially among those with previous lung transplant (OR 11.16, 95%‐CI 2.37–52.57, *p* < 0.01). CF is associated with lower CRC‐free survival as illustrated in Figure 4. Mean age at cancer diagnosis was younger in CF cohort (40.3 vs. 54.5 years).

Tumors were located in cecum in three patients, ascending colon in three, transverse colon in two, and sigmoid/descending colon in five. One patient had three synchronous tumors found during same colonoscopy. Previous lung transplant and poor bowel preparation were predictors of CRC on univariate analysis (Table 5). Seven CF patients with CRC underwent surgical resection, while five patients had metastatic cancer at diagnosis.

**TABLE 5:** Logistic regression analysis of predictors for CRC in CF cohort.

Variables	Univariate analysis	
	OR (95% CI)	*p*
Age (per decade)	1.23 (0.73–2.09)	0.45
Gender (reference: male)	1.45 (0.43–4.96)	0.55
CF mutation (reference: heterozygous)	—	
Lung transplant (reference: no previous transplant)	4.13 (1.09–15.66)	0.04
Time since transplantation (years)	1.03 (0.93–1.15)	0.56
Family history of CRC	2.02 (0.42–9.83)	0.38
Colonoscopy indication (reference: screen for CRC only without symptoms)		
Low‐risk symptoms only	4.25 (0.74–24.42)	0.11
One higher risk symptom/indication	1.34 (0.25–7.08)	0.73
Two or more higher risk symptoms/indications	5.67 (0.45–71.96)	0.18
Presence of GI symptoms	2.63 (0.81–8.54)	0.11
ASA (reference: ASA 2)		
ASA 3	1.07 (0.22–5.21)	0.93
ASA 4	3.07 (0.39–23.69)	0.28
Quality of bowel preparation (reference: good/excellent)		
Average	0.40 (0.04–3.93)	0.43
Poor	4.44 (1.06–18.57)	0.04

Abbreviations: ASA: American Society of Anesthesiologists physical assessment Score; CF: cystic fibrosis; CRC: colorectal cancer; OR: unadjusted odds ratio.

### 3.4 Surveillance Outcomes

In CF cohort, 148/245 (60.4%) patients underwent surveillance colonoscopy at mean surveillance interval of 3.3 years during study period, with median two surveillance colonoscopies performed. This was higher than the control cohort (56/245, 22.9%, OR 5.14, 95%‐CI 3.48–7.63, *p* < 0.01); 65.8% of CF patients with neoplastic lesions detected at initial colonoscopy underwent further surveillance. Seventy‐four (70.5%) of surveillance colonoscopies were performed within 3 years as recommended in consensus statements published by Hadjiliadis et al. [14]. Forty‐four out of 74 (59.5%) of surveillance colonoscopies performed within 3 years of preceding colonoscopy that had detected neoplastic lesions found further lesions, of which 9/74 (12.1%) were advanced lesions and 1/74 (1.4%) detected CRC. The remaining patients with CRC detected during surveillance were diagnosed after median 4 years from preceding colonoscopy.

Two hundred out of 354 (56.5%) colonoscopies with no neoplastic lesions detected had further surveillance colonoscopy; 77.5% were performed within 5 years as recommended by consensus statements published by Hadjiliadis et al. [14]. Among these, 34.7% detected neoplastic lesions, of which 3.5% were advanced lesions. No cases of CRC were detected within 5 years of a preceding colonoscopy that had no neoplastic lesions detected.

Death from all causes occurred in 29 patients in the CF cohort and 7 patients in the control cohort. CRC‐related death occurred in six patients in the CF cohort and one patient in the control cohort.

## 4 Discussion

Our study has demonstrated that patients with CF are at significantly higher risk of colorectal neoplasia and CRC compared to age‐ and gender‐matched controls, especially among those with previous lung transplantation. Other studies have found similar results; Gory et al. [9] published a similar study from our site finding a 32% rate of advanced adenoma and 10% rate of CRC in 50 CF patients, corresponding to a 10‐fold increased risk of CRC compared to 100 matched controls. The higher incidence of advanced lesions compared to our study may result from inclusion of lesion multiplicity as part of their definition for advanced lesions, which was not included in ours. Yamada et al. in a meta‐analysis of 99 925 patients similarly showed higher CRC risk (OR 10.91) in CF patients [16]. Hegagi et al. demonstrated a higher rate of adenoma detection in a prospective study (RR 9.29) in CF patients but their study was insufficiently powered to show differences in polyp histology or CRC incidence [17]. Niccum et al., in a prospective study of 88 patients, found > 50% incidence of adenomatous polyps, 25% advanced adenomas, and 3% CRC [18].

The incidence of CRC has previously been shown to be disproportionately higher in CF; Maisonneuve et al. demonstrated elevated risk of gastrointestinal cancer (SIR 3.5) [13]. The mechanisms behind this are not fully understood; mutations in the CFTR gene causing chronic inflammation, dysregulated immune responses, and loss of intestinal barrier integrity in CF create a pro‐carcinogenic microenvironment [19]. Additionally, CF patients often exhibit intestinal dysbiosis, characterized by an imbalance of beneficial bacteria species such as Bifidobacterium and lactobacillus with potentially harmful species including Enterobacteriaceae and *Pseudomonas aeruginosa* , contributing to inflammation, impaired intestinal barrier function, and increased susceptibility to CRC [19, 20, 21].

Effectiveness of CRC screening in CF may be impaired by difficulty in achieving adequate bowel clearance, which is unsurprising in patients with CF due to thickened intestinal secretions and slow colonic transit [22, 23, 24, 25]. In our study, poor bowel preparation was more frequently seen in CF compared to the control cohort; therefore, potentially the true incidence of colorectal neoplasia in our CF cohort may have been underestimated in spite of the intensive bowel preparation regimen. Matson showed in a nonrandomized single‐center cohort study that a modified bowel preparation regimen for CF patients had significantly better rates of good/excellent preparation quality (50% vs. 25.9%, < 0.01), associated with greater adenoma detection at initial colonoscopy [26]. Other studies have shown split‐dose bowel regimens to be superior to same‐day bowel prep regimens for polyp detection [27]. However, there are no high‐quality randomized studies to determine the optimal bowel preparation regimen for CF. Therefore, patient education, emphasis on adherence, and structured recall systems for early rescheduling of colonoscopies following poor preparation remain vital for optimizing lesion detection.

Our study identified previous lung transplantation and increasing age as predictors of colorectal neoplasia, but not of advanced lesions, on multivariate analysis. Increased incidence of colorectal neoplasia in those with previous lung transplantation may potentially be attributable not only to immunosuppression but also to those requiring transplantation likely having more severe underlying disease phenotype driving greater inflammation and therefore carcinogenesis independent of immunosuppression. Interestingly, delF508 homozygosity was predictive on univariate analysis for advanced lesions. However, CF mutation genotype was not identified in 37% of CF patients in our study, all being among patients with previous lung transplantation referred from other centers whose pretransplant medical records were not accessible by study investigators. CF mutation has not been clearly identified in other studies to be an independent predictor for CRC; Maisonneuve et al. reported that, among patients homozygous for the delf508 mutation, the SIR for all GI cancers was 5.2 (95%‐CI 1.9–11.3), compared with an SIR of 2.5 in patients with non‐delF508 mutations [28]. In contrast, Niccum et al. identified delF508 homozygosity as an independent predictor of all colonic polyps, but not advanced polyps, on multivariate analysis. In the absence of clear independent predictors for advanced lesions or CRC identified by logistic regression in this study, we believe a risk prediction model for CRC in CF prior to colonoscopy is not yet feasible and therefore screening and surveillance guidelines should be standardized across all CF patients.

As mentioned previously, consensus statements recommending CRC screening via colonoscopy commence at age 40, with ≤ 3 yearly surveillance intervals following detection of neoplastic lesions and 5 yearly intervals with no lesions detected [14]. However, these recommendations are based on a cost–benefit analysis performed by Gini et al., which showed that screening from age 40 at intervals of no more than 5 years in patients with CF who have not undergone transplantation, and from age 30 in those with a previous transplant, would prevent 79% of CRC‐related deaths [29]. Studies assessing optimal surveillance intervals or effectiveness of surveillance for colorectal neoplasia in CF are few and small in sample size; in a study by Billings et al. of 45 CF patients, 7 underwent 14 surveillance colonoscopies, of which 12 colonoscopies identified new adenomatous polyps [30]. In the study by Niccum et al., 81% of patients with adenomatous polyps detected on the initial surveillance colonoscopy continued to have lesions on surveillance colonoscopy performed after a median interval of 1–2 years [18].

A major strength of our study is that compared to other studies, we have by far the largest single‐center cohort of CF patients (*n* = 245) that have undergone colonoscopy. In our study, the incidence of CRC among asymptomatic CF patients undergoing screening colonoscopy is higher compared to the incidence of definite CRC among the general Australian population undergoing CRC screening via the National Bowel Cancer Screening Program in 2022 (5.5% vs. 0.6%, OR 9.96, 95%‐CI 3.61–27.47, *p* < 0.01) [31]. Additionally, the younger mean age at CRC diagnosis for the CF cohort of 40.3 years, and 40.5 years among the four asymptomatic patients, compared to the control cohort suggests earlier onset of CRC in patients with CF. This supports consensus statements by Hadjiliadis et al. recommending earlier screening for CRC among patients with CF, though further study may be required to determine the optimal age for commencing CRC screening given the comparatively higher CRC incidence in our CF cohort at age 40. In our study, the relatively high proportion (59.5%) of surveillance colonoscopies performed after a median of 3.3 years finding more neoplastic lesions, including four cases of CRC, reinforces the importance of regular surveillance in CF. Further study is required to determine optimal surveillance intervals.

This study has some limitations; our CF cohort is a heterogeneous cohort undergoing elective colonoscopy for a variety of different indications; therefore, generalizing results to determine optimal timing for screening and surveillance in asymptomatic individuals remains difficult. Our CF cohort is also younger in mean age than the general Australian population, which up until recently had commenced CRC screening at age 50; therefore, there is an inherent bias by indication whereby there is a much lower proportion of the control cohort undergoing screening colonoscopy without having symptoms or other indications compared to the CF cohort. This likely reflects difficulties in the matching process for the control group. Additionally, the significantly higher number of surveillance colonoscopies performed in CF compared to the control cohort does create bias by providing more opportunity for neoplastic lesion detection; therefore, the incidence of colorectal neoplastic lesions may be underestimated in the control cohort.

Future directions of study include prospective randomized studies assessing the adequacy of our multiday split‐bowel prep regimen protocol compared to standard bowel regimen protocol in patients with CF, as well as further longitudinal studies assessing outcomes in surveillance in CF. Additionally, of particular interest would be if Trikafta (elexacaftor/tezacaftor/ivacaftor), a chloride channel opener and CFTR modulation combination treatment approved for use in Australia in 2021, can reduce CRC risk in CF, given its benefit in respiratory symptoms and function [32].

## 5 Conclusion

Our study demonstrates that the risk of colorectal neoplasia and cancer is increased in patients with CF and can occur at a younger age than the general population. Detection of colorectal lesions is affected by higher rates of poor bowel preparation; therefore, bowel preparation optimization is recommended in CF patients. Close collaboration between respiratory and gastroenterology teams is necessary to identify patients with CF suitable to commence screening for CRC and maintain compliance with ongoing surveillance, given the high incidence of continued neoplastic lesions.

## Funding

The authors have nothing to report.

## Ethics Statement

This study was conducted in accordance with the Helsinki Declaration of 1975 and approved by the Ethics Committee of the Alfred Hospital (Project no. 359/24).

## Consent

The authors have nothing to report.

## Conflicts of Interest

The authors declare no conflicts of interest.

## Supporting information


**Table S1:** Logistic regression analysis of predictors for advanced lesions in CF cohort.
**Figure S1:** Number of elective colonoscopies performed in CF patients per year from 2010 to 2024.
**Figure S2:** Proportion of total colonoscopies with detected neoplastic lesions, advanced lesions, and cancer compared among CF cohort with lung transplant, without lung transplant, and control cohort.

## Data Availability

The data that support the findings of this study are available from the corresponding author upon reasonable request.
