# Supplementary material for: Investigating the Association Between Cystic Fibrosis and Colorectal Neoplasia: A Matched Case–Control Study
Source: JGH Open. 2026 Jul 14;10(7):e70440. doi: 10.1002/jgh3.70440 (PMC13366771; doi:10.1002/jgh3.70440)
Supplement: Supplementary file 1 — Table S1: Logistic regression analysis of predictors for advanced lesions in CF cohort. Figure S1: Number of elective colonoscopies performed in CF patients per year from 2010 to 2024. Figure S2: Proportion of total colonoscopies with detected neoplastic lesions, advanced lesions, and cancer compared among CF cohort with lung transplant, without lung transplant, and control cohort. [file JGH3-10-e70440-s001.docx]

# Supplementary Material

| **Supplementary Table 1: Logistic regression analysis of predictors for advanced lesions in CF cohort** | | | | | |
| --- | --- | --- | --- | --- | --- |
| Variables | | Univariate analysis | | Multivariate analysis | |
|  |  | OR (95% CI) | *P* value | OR (95% CI) | *P* value |
| Age (per decade) | | 1.19 (0.87-1.64) | 0.28 |  |  |
| Gender (reference male) | | 0.75 (0.38-1.48) | 0.41 |  |  |
| CF mutation (reference heterozygous) | | 17.42 (2.25-134.6) | 0.01 |  |  |
| Lung transplant (reference NO transplant) | | 1.73 (0.87-3.41) | 0.12 |  |  |
| Time since transplantation (years) | | 0.98 (0.90-1.05) | 0.52 |  |  |
| Family history of CRC | | 1.95 (0.72-5.30) | 0.19 |  |  |
| Colonoscopy indication (reference screen for CRC only without symptoms) | Low risk symptoms only | 1.94 (0.68-5.55) | 0.22 |  |  |
|  | 1 higher risk symptom/indication | 1.21 (0.52-2.82) | 0.66 |  |  |
|  | 2 or more higher risk symptoms/indications | 5.08 (0.97-26.53) | 0.05 |  |  |
| Presence of GI symptoms | | 1.58 (0.79-3.14) | 0.19 |  |  |
| ASA (reference ASA 2) | ASA 3 | 1.79 (0.66-4.88) | 0.26 |  |  |
|  | ASA 4 | 2.65 (0.62-11.32) | 0.19 |  |  |
| Quality of bowel preparation (reference good/excellent) | Average | 0.88 (0.38-2.04) | 0.77 |  |  |
|  | Poor | 1.61 (0.68-3.83) | 0.28 |  |  |

Supplementary Figure 1. Number of elective colonoscopies performed in CF patients per year from 2010-2024

Supplementary Figure 2. Proportion of total colonoscopies with detected neoplastic lesions, advanced lesions and cancer compared amongst CF cohort with lung transplant, without lung transplant and control cohort
